# The job demands–resources model and safety behavior in construction work: a systematic review and meta-analysis of six dual-process paths

**DOI:** 10.3389/fpubh.2026.1938727

**Published:** 2026-09-23

**Authors:** Dan Wang, Yunyun Qin

**Affiliations:** School of Business Administration, Liaoning Technical University, Huludao, China

**Keywords:** China, construction workers, extreme heat, job demands-resources model, meta-analysis, safety behavior

## Abstract

**Background:**

Construction workers’ safety behavior is central to accident prevention, yet the psychological paths linking work characteristics to safety behavior have not been transparently synthesized in the construction industry.

**Methods:**

This systematic review and meta-analysis examined the six-path Job Demands–Resources (JD-R) model and evaluated extreme heat as a possible contextual boundary condition. Following a revision-stage construct and dependency audit, we synthesized 194 zero-order correlations from 63 reports representing 64 independent samples (*N* = 22,846) with random-effects models.

**Results:**

Job demands were positively associated with strain (*r* = 0.321), job resources with motivation (*r* = 0.578), and motivation with safety behavior (*r* = 0.439). The pooled strain–safety behavior association was small and inconclusive (*r* = −0.060; I^2^ = 99.0%). The two cross-links were based on few samples and are therefore descriptive only: demands–motivation *r* = −0.124 (*k* = 5) and resources-strain *r* = −0.294 (*k* = 3). No evidence of heat-proxy subgroup differences was found for resources–motivation (*p* = 0.628) or motivation–safety behavior (*p* = 0.463), while demands–strain and strain–safety behavior could not be tested because no eligible high-heat samples remained.

**Conclusion:**

These results indicate insufficient evidence to establish heat moderation, rather than evidence that heat is irrelevant. Most evidence was cross-sectional, and heterogeneity was substantial. Construction managers should strengthen supervisory support, safety climate, and safety motivation while also managing heat exposure directly through exposure monitoring, hydration, shade, and work–rest controls.

## 1 Introduction

Construction remains a high-risk industry in China. National accident summaries indicate that 6,005 fatal construction accidents caused 7,275 deaths during 2010–2019, while analyses of Ministry of Housing and Urban–Rural Development reports identify falls, outdoor exposure, labor-intensive work, and workforce mobility as persistent risk factors (1, 2). Unsafe acts—including failure to use protective equipment, procedural deviations, and risk-taking under time pressure—are important proximal contributors to site accidents. The practical question is therefore not only which conditions are hazardous but also which work characteristics managers can modify to support safer behavior. The Job Demands–Resources (JD-R) model provides a parsimonious framework: job demands require sustained effort and can produce strain, whereas job resources facilitate goal attainment and can foster motivation (3, 4).

The JD-R model has been widely applied to safety outcomes. Meta-analytic evidence indicates that job demands are associated with burnout and adverse safety outcomes, whereas job resources support safety compliance and participation through engagement and safety motivation (5, 6). Longitudinal evidence further shows that safety climate predicts later safety behavior through safety motivation (7). Nevertheless, the majority of JD-R safety syntheses span multiple industries and treat physical working conditions as background context. Construction work in China offers a theoretically informative setting because construction activities are frequently conducted outdoors, are labor-intensive and team-dependent, and are subject to marked seasonal and regional variation.

The present contribution is not the claim that heat creates a seventh JD-R construct. Conceptually, extreme heat is a physical job demand because it imposes physiological and cognitive costs. Analytically, however, heat may also operate as a boundary condition: it may change the strength of the associations through which other demands and resources translate into strain, motivation, and safety behavior. Distinguishing these two roles avoids treating a contextual exposure and a focal psychological predictor as interchangeable. The primary theoretical question is whether the dual processes generalize across construction work samples, including the substantial body of evidence from China; the secondary, evidence-mapping question is whether the available data support heat-contingent path strengths.

Heat plausibly alters JD-R processes through resource depletion. Thermoregulation and thermal discomfort compete for attention and executive control, potentially strengthening the demands–strain association and impeding the conversion of motivation into protective action (8, 9). This reasoning is compatible with conservation of resources theory (10), but the primary psychological evidence is limited. Long-term acclimatization, self-reported heat proxies, and regional confounding may attenuate or distort subgroup differences. The moderation hypotheses are therefore theory-driven but necessarily provisional.

Where the high-heat subgroup contains fewer than two independent samples, the corresponding moderation hypothesis is reported as not testable. Non-significant subgroup tests are interpreted as no detected moderation under the available evidence, not as proof of the absence of a heat effect.

This meta-analysis makes three contributions. First, it evaluates all six JD-R paths within construction work while separating the report, sample, and effect-size levels of the evidence. Second, it clarifies construct boundaries by distinguishing safety compliance and participation from behavioral intention, incident outcomes, and correlations among safety outcomes. Third, it treats heat simultaneously as a physical demand and a candidate contextual boundary condition, thereby mapping where evidence is sufficient for analysis and where it is not. China remains an important application context, but the synthesis is not restricted to Chinese samples. The revision also reconciles the inherited corpus with newly verified studies and provides an auditable decision log, sample crosswalk, and analysis plan.

These findings therefore bear on boundary conditions rather than a new causal law: Successful moderation would show that the operation of established JD-R processes depends on physical context, whereas a non-significant test with sparse heat data cannot establish invariance.

## 2 Theoretical framework and hypotheses

### 2.1 The dual process of the JD-R model

Job demands require sustained effort and carry costs, whereas job resources help achieve goals, reduce demands, or stimulate growth. The JD-R model describes two relatively independent processes (4). We first outline these processes and derive the six main-effect hypotheses (H1–H6).

#### 2.1.1 Health-impairment process

Chronic demands—time pressure, role conflict, and workload—drain workers’ limited resources, producing strain (burnout, exhaustion, fatigue, and negative affect). This process is consistent with the resource-depletion perspective: individuals strive to retain valued resources, and resource loss is psychologically more impactful than gain (10). Strained workers have less cognitive capacity to monitor hazards, resist risky shortcuts, or comply with protective procedures. Meta-analytic evidence confirms that job demands are positively associated with strain, and strain negatively predicts safety behavior (5, 6). It is hypothesized that demands will increase strain (H1) and that this strain will reduce safety behavior (H2).

#### 2.1.2 Motivational process

Parallel to the health-impairment path, the motivational process operates through a different mechanism. Resources—supportive leadership, safety climate, and psychological capital—enhance safety motivation by satisfying basic psychological needs (autonomy, competence, and relatedness) (11). Internalized safety motivation drives sustained compliance and participation (7). Recent meta-analyses reaffirm that job resources are positively associated with safety motivation and that motivation predicts safety behavior (5, 6). It is hypothesized that resources will increase motivation (H3) and that this motivation will increase safety behavior (H4).

#### 2.1.3 Cross-links

H5 predicts a negative association between demands and motivation because sustained hindrance demands consume resources needed for motivated action. H6 predicts a negative association between resources and strain because resources can buffer resource loss (4). Challenge demands may not follow the same pattern as hindrance demands (12), so pooled cross-links are expected to be heterogeneous and are interpreted cautiously when based on few samples.

These six hypotheses describe how the work environment, on average, relates to safety behavior. However, physical work conditions—particularly extreme heat—may alter the strength of these relationships. We turn to this possibility next.

### 2.2 Extreme heat as a physical job demand

Extreme heat is conceptualized here in two non-equivalent ways: as a physical demand in its own right and, for the subgroup tests, as a contextual condition that may change the strength of other JD-R paths. The moderation analyses use a coarse season/region indicator because objective wet-bulb globe temperature (WBGT) was rarely reported. Consequently, hypotheses H7–H10 test a boundary-condition proposition using an imperfect proxy; they do not estimate the direct causal effect of temperature.

H7 (heat strengthens demands→strain): Heat imposes its own physiological load: thermoregulation competes for attentional and executive resources, reducing the cognitive capacity available for coping with psychological job demands (8). When time pressure, role conflict, or workload are added to an already heat-stressed system, the combined resource drain may exceed the sum of the two separately. The positive demands–strain association is therefore predicted to be stronger under high heat (H7).

H8 (heat weakens resources→motivation): Job resources operate partly by satisfying basic psychological needs (autonomy, competence, and relatedness). However, heat-induced fatigue narrows attention and increases focus on somatic discomfort, reducing workers’ receptivity to supervisory support or safety messaging (9). Heat may override positive cues: thermal discomfort dominates the affective state, swamping the motivational benefits ordinarily provided by resources. The positive resource–motivation association is predicted to be weaker under high heat (H8).

H9 (heat strengthens strain → safety behavior): Strain already degrades safety behavior by depleting self-regulatory resources. Heat adds another layer: elevated core temperature and fatigue impair executive functions—working memory, sustained attention, and cognitive flexibility (13). This impairment reduces workers’ capacity to override impulsive shortcuts (e.g., removing protective gear to cool down and skipping rest breaks). The negative strain–safety behavior association is predicted to be stronger (more negative) under high heat (H9).

H10 (heat weakens motivation → safety behavior): Safety motivation is necessary but not sufficient. Translating intention into action requires cognitive resources: planning, self-monitoring, and effortful initiation of protective behaviors. Heat stress directly depletes these executive resources (9, 13), creating a motivation–behavior gap: workers know what should be done and want to do it, but lack the cognitive bandwidth to execute. The positive motivation–safety behavior association is predicted to be weaker under high heat (H10).

When the high-heat subgroup contains fewer than two independent studies, the corresponding hypothesis is reported as not testable rather than resolved.

## 3 Materials and methods

### 3.1 Protocol and transparency

The review was not prospectively preregistered because it originated as a secondary analysis of an existing coding workbook rather than a newly initiated systematic review. The available files do not establish that every original inclusion and analysis decision preceded the original extraction, and we do not claim that they did. Before the present revision-stage reanalysis, we froze a dated analysis amendment (19 August 2026; Supplementary File 1) specifying construct mappings, sample clustering, outlier handling, and sensitivity analyses. It is labeled retrospective and is intended to reduce flexibility in the revision, not to imply that the original review was preregistered. The revision follows the Preferred Reporting Items for Systematic reviews and Meta-Analyses (PRISMA) 2020 (14) as a reporting framework where the inherited records permit; unavailable historical identification and screening fields are marked as unavailable rather than reconstructed without evidence.

### 3.2 Literature base and search

The study began as a secondary reanalysis of an inherited coding corpus. The coding workbook contained 117 report blocks and 121 report-sample records derived from searches of the Web of Science, Scopus, China National Knowledge Infrastructure (CNKI), and Wanfang Data databases. English- and Chinese-language reports were eligible, and no geographic restriction was applied; China is a prominent application context, not the sole source of samples. The original database export files, exact execution dates, and record-level identification counts were not preserved; we therefore do not retrospectively invent those quantities. Supplementary Table S1 provides database-specific reconstructed search strings, the language scope, and the available corpus inventory. Of the 117 inherited report blocks, 69 were represented in the surviving 255-row inherited effect-size file; 48 were not represented, and their original report-level dispositions were not fully archived. Revision-stage adjudication retained eligible primary six-path effects from 60 of the 69 represented reports and found no eligible primary contribution in nine reports. The 60 retained inherited reports comprised 52 pre-update reports and eight 2025–2026 reports already represented in the analytical file. Three further reports—Long et al. (15), Li et al. (16), and Mo and Jiang (17)—contributed 20 eligible correlations after full-text verification. The inherited analytical file separately contains a different report labeled Li et al. (25; *N* = 407), whereas the newly verified AI-adoption report has *N* = 425; Supplementary Data Files S1–S3 distinguish them by the unique sample identifiers RS_Li, 2026 and LI_2026_AI. The final quantitative synthesis therefore contains 63 reports representing 64 independent samples.

The reconstructed search syntax used construction worker terms, safety behavior outcomes, and JD-R demand/resource, strain, and motivation terms. English- and Chinese-language publications were eligible; no other language was searched, and no geographic restriction was applied. Because the historical execution dates and database result exports are unavailable, the exact strings are supplied for reproducibility of the next update rather than represented as contemporaneously archived original queries.

### 3.3 Inclusion and exclusion criteria

Eligible effects were individual-level zero-order correlations among job demands, job resources, strain, motivation, and safety behavior in construction workers. Safety compliance and safety participation were treated as two facets of safety behavior. Unsafe-behavior coefficients were sign-reversed so that higher values consistently indicated safer behavior. Behavioral intention, perceived behavioral control, incident/near-miss outcomes, and correlations among two safety outcomes were excluded from the primary synthesis unless the construct could be defensibly mapped to motivation or behavior from the original scale description. Behavioral-intention records were retained for sensitivity analysis. Ecological heat–injury associations and heat-as-predictor effects were excluded because they do not estimate an individual-level JD-R path.

For construct consistency, compliance and participation were pooled as behavioral facets; unsafe behavior was classified as sign-oriented; safety performance was retained only when the scale was behavior-based; safety voice was treated as participation when it was itself the outcome, but safety–outcome intercorrelations were not treated as JD-R paths. These decisions were applied before the final reanalysis and logged row by row.

Records were also excluded when the variable pair did not instantiate any of the six paths, when only adjusted path coefficients were available without a zero-order correlation, or when the source could not be verified. Two near-perfect correlations (|*r*| ≥ 0.99) were quarantined from the primary model pending source verification; a broader influential-effect sensitivity analysis excluded correlations with |*r*| > 0.85. Supplementary Data File S1 contains the 255 inherited records plus the 20 file-verified additions, with the decision, reconstructed path, orientation, source, and reason for each row.

### 3.4 Handling of non-independent effect sizes

Exact duplicate rows were removed first. Multiple correlations from the same underlying sample and path were transformed to Fisher’s *z* and averaged before meta-analysis. Independent samples reported within one article were separated using the coding workbook’s sample identifiers; publications sharing a global sample identifier were clustered as one underlying sample. This procedure yielded 64 independent samples in the final synthesis. Within each sample-path cell, *N* was the largest applicable sample size, and the overall participant count was calculated by summing the maximum *N* once for each underlying sample.

### 3.5 Construct path adjudication and effect orientation

Path assignment was rebuilt from the actual x and y constructs rather than inherited H labels. Correlations were treated as symmetric when the variables were listed in reverse order. Safety knowledge, ability, AI usefulness, adaptability, and work support were mapped as resources when their scale content represented capacity or support; safety attitude, responsibility, relational energy, thriving, and safety consciousness were mapped as motivational constructs; fatigue, burnout, anxiety, depression, symptoms, and cognitive dissonance were mapped as strain. According to Li et al. (16), this mapping followed the original construct definitions and measures: AI adoption indexed the extent to which workers used and spent time working with AI and was treated as a work demand; perceived AI usefulness reflected task-enabling support, and adaptability reflected the capacity to adjust to changing work requirements, so both were placed on the resource side; a sense of safety responsibility and relational energy were treated as motivational states; and extra-role safety behavior was coded as safety participation. Cognitive dissonance was classified as strain because the verified source defines it as an aversive affective state that consumes psychological resources. Ambiguous mappings were retained in the inclusive primary model only when they were defensible on the basis of theory and scale content and were removed in a restricted sensitivity model.

### 3.6 Statistical analysis

Correlations were transformed to Fisher’s z with a sampling variance of 1/(N – 3). DerSimonian-Laird random-effects models were used for the primary estimates; pooled values and 95% confidence intervals were back-transformed to r. Heterogeneity was summarized with Q, Δz, and τ². Heat-proxy subgroup differences were evaluated only when each subgroup contained at least two independent samples. Because H5 and H6 were based on only five and three independent samples, respectively, their pooled estimates are interpreted descriptively and are not used to make strong hypothesis claims.

The primary analysis used zero-order correlations. Standardized regression coefficients were not mixed with correlations in the primary pool. Sensitivity analyses compared the inclusive mapping with a restricted rule-based construct mapping, removed influential correlations with |*r*| > 0.85, and treated safety behavior intention as a proxy outcome. Construct consistency was additionally examined with dependence-robust comparisons of safety compliance and participation, with observations clustered by underlying sample.

Potential heterogeneity sources were audited systematically. The heat group and safety behavior facets were testable. Formal occupation, measurement scale, and design meta-regressions were not estimable with acceptable coverage: occupation labels were highly fragmented, scale names were absent from the inherited workbook, and only two H4 samples were longitudinal (all other coded designs were cross-sectional or unknown). The region was similarly unbalanced, with only one Hong Kong sample in H3 and H4. These limitations are reported as missing moderator information rather than interpreted as evidence that these features do not matter.

Analyses were implemented in reproducible Python code, with the compliance–participation sensitivity analysis estimated using small-sample CR2 robust variance procedures in R. DerSimonian-Laird random-effects estimation and I^2^ followed established methods (18, 19). Values are reported to three decimals, and *p*-values less than 0.001 are shown as such. Egger regression analysis (20) was limited to paths with *k* ≥ 10. A prospective preregistration was not conducted; the dated revision-stage analysis plan in the supplement documents the decisions made before the present reanalysis and is explicitly labeled as retrospective, not as a substitute for prospective registration.

### 3.7 Study quality and risk-of-bias considerations

A formal report-level risk-of-bias assessment was not preserved for the inherited corpus, and full texts were not available for every inherited report during the revision. The recoverable design coding shows that 102 of 121 inherited report-sample records were cross-sectional, five were longitudinal, and 14 had no archived design label. The synthesis is therefore interpreted as associational and is vulnerable to common-method, temporal-order, and selective-reporting bias. Confidence is further reduced where k is small or heterogeneity is extreme. A future prospectively registered update should retrieve every full text and apply a design-appropriate risk-of-bias tool independently by two reviewers.

## 4 Results

### 4.1 Evidence flow and descriptive statistics

Figure 1 presents the recoverable report-level corpus reconciliation. The inherited workbook contained 117 report blocks. Sixty-nine report blocks were represented in the surviving 255-row inherited effect-size file, and 48 were not represented, with the original report-level dispositions of those 48 not being fully archived. Revision-stage adjudication retained eligible primary six-path effects from 60 of the 69 represented reports and found no eligible primary contribution in nine reports. Seven external candidate reports were assessed from supplied full texts during revision; three contributed eligible new correlations, and four did not contribute to the primary six-path synthesis. The final primary synthesis therefore comprised 63 reports, representing 64 independent samples and 22,846 participants. The effect-size audit consisted of 343 coded entries, with 275 after removing 68 exact duplicates, 255 after 20 original eligibility exclusions, 174 inherited correlations after construct/path adjudication, and 194 after 20 file-verified additions. Across paths, k ranged from 3 to 47. Historical database identification, deduplication, title/abstract screening, and retrieval counts remain unavailable and are shown as such rather than reconstructed.

**Figure 1:**
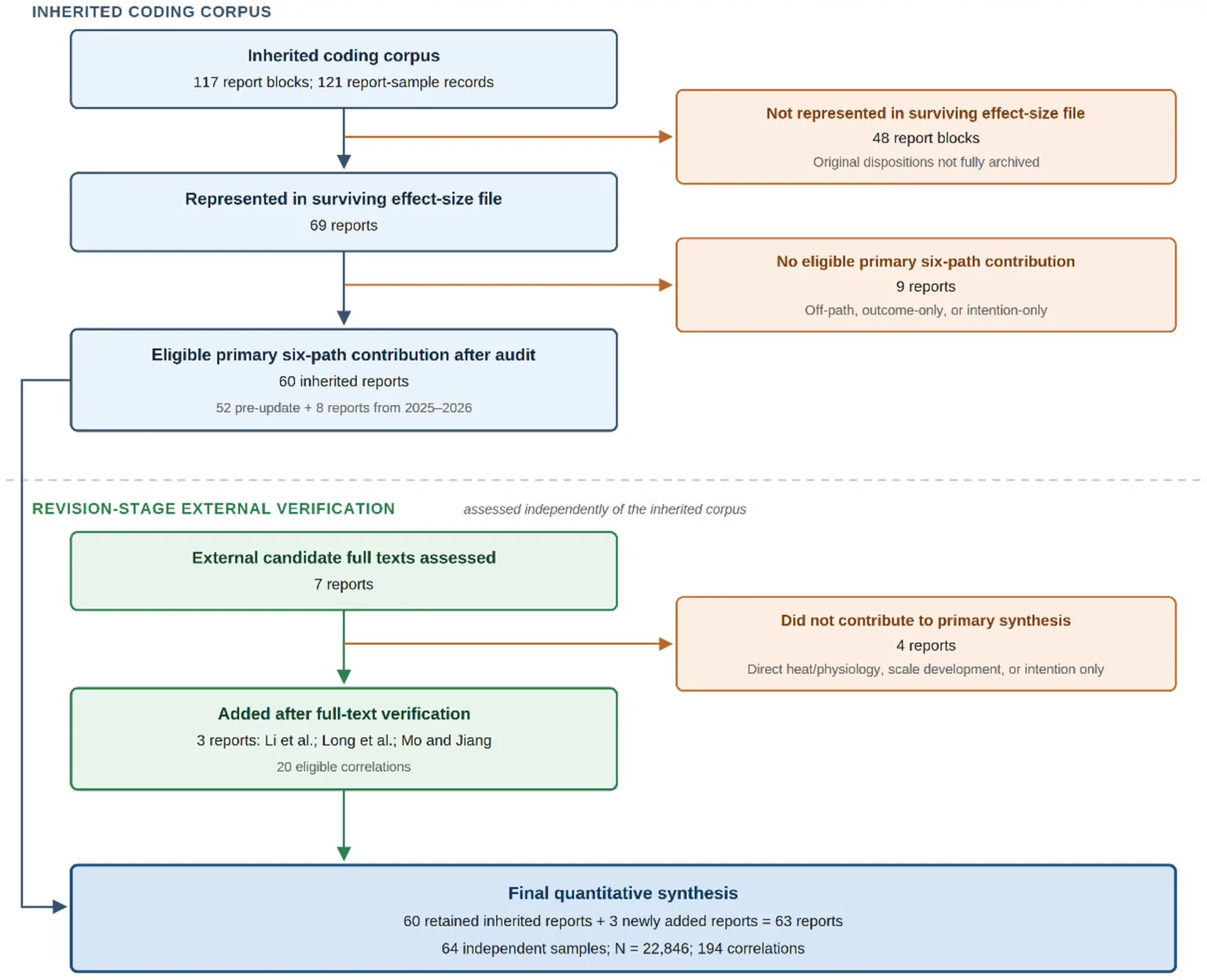
Flowchart of the study selection process.

### 4.2 Main effect paths (H1–H6)

Table 1 reports the random-effects estimates. Job demands were positively associated with strain (H1: *r* = 0.321, 95% CI [0.037, 0.558], *k* = 6, *p* = 0.027). Job resources were positively associated with motivation (H3: *r* = 0.578, 95% CI [0.467, 0.672], *k* = 30, *p* < 0.001). Motivation was positively associated with safety behavior (H4: *r* = 0.439, 95% CI [0.287, 0.569], *k* = 47, *p* < 0.001). Heterogeneity was high for all three paths (I^2^ = 98.2–99.2%).

**Table 1:** Random-effects estimates for the six JD-R paths after construct and dependency adjudication.

Path (H)	*k*	*N*	*r*	95% CI	*p*	*Q*	I^2^ (%)	τ^2^
H1 Demands → Strain	6	2,532	0.321	[0.037, 0.558]	0.027	274.729	98.2	0.1342
H2 Strain → Safety Behavior	9	3,195	−0.060	[−0.390, 0.284]	0.739	803.836	99.0	0.2867
H3 Resources → Motivation	30	10,594	0.578	[0.467, 0.672]	<0.001	1858.742	98.4	0.1810
H4 Motivation → Safety Behavior	47	16,351	0.439	[0.287, 0.569]	<0.001	5920.078	99.2	0.3716
H5 Demands → Motivation	5	1,551	−0.124	[−0.428, 0.206]	0.464	171.119	97.7	0.1406
H6 Resources → Strain	3	1,458	−0.294	[−0.463, −0.103]	0.003	26.298	92.4	0.0283

The pooled strain–safety behavior association was close to zero and inconclusive (H2: *r* = −0.060, 95% CI [−0.390, 0.284], *k* = 9, *p* = 0.739; I^2^ = 99.0%). The interval includes both a practically negative association and a positive association. This result is therefore interpreted as reflecting unresolved heterogeneity, not as evidence that strain has no relation to safety behavior.

The motivational sequence received the clearest support: resources were associated with motivation and motivation with safety behavior. These estimates remained positive under the restricted rule-based construct mapping (H3 *r* = 0.580; H4 *r* = 0.433) and when correlations with |*r*| > 0.85 were removed (H3 *r* = 0.474; H4 *r* = 0.383).

The cross-links were based on small evidence bases. The demands-motivation association was negative but imprecise (H5: *r* = −0.124, 95% CI [−0.428, 0.206], *k* = 5, *p* = 0.464). The resources-strain association was negative (H6: *r* = −0.294, 95% CI [−0.463, −0.103], *k* = 3), but *k* = 3 is insufficient for stable between-study variance estimation. H5 and H6 are therefore descriptive and are not treated as confirmed or rejected hypotheses (Figure 2).

**Figure 2:**
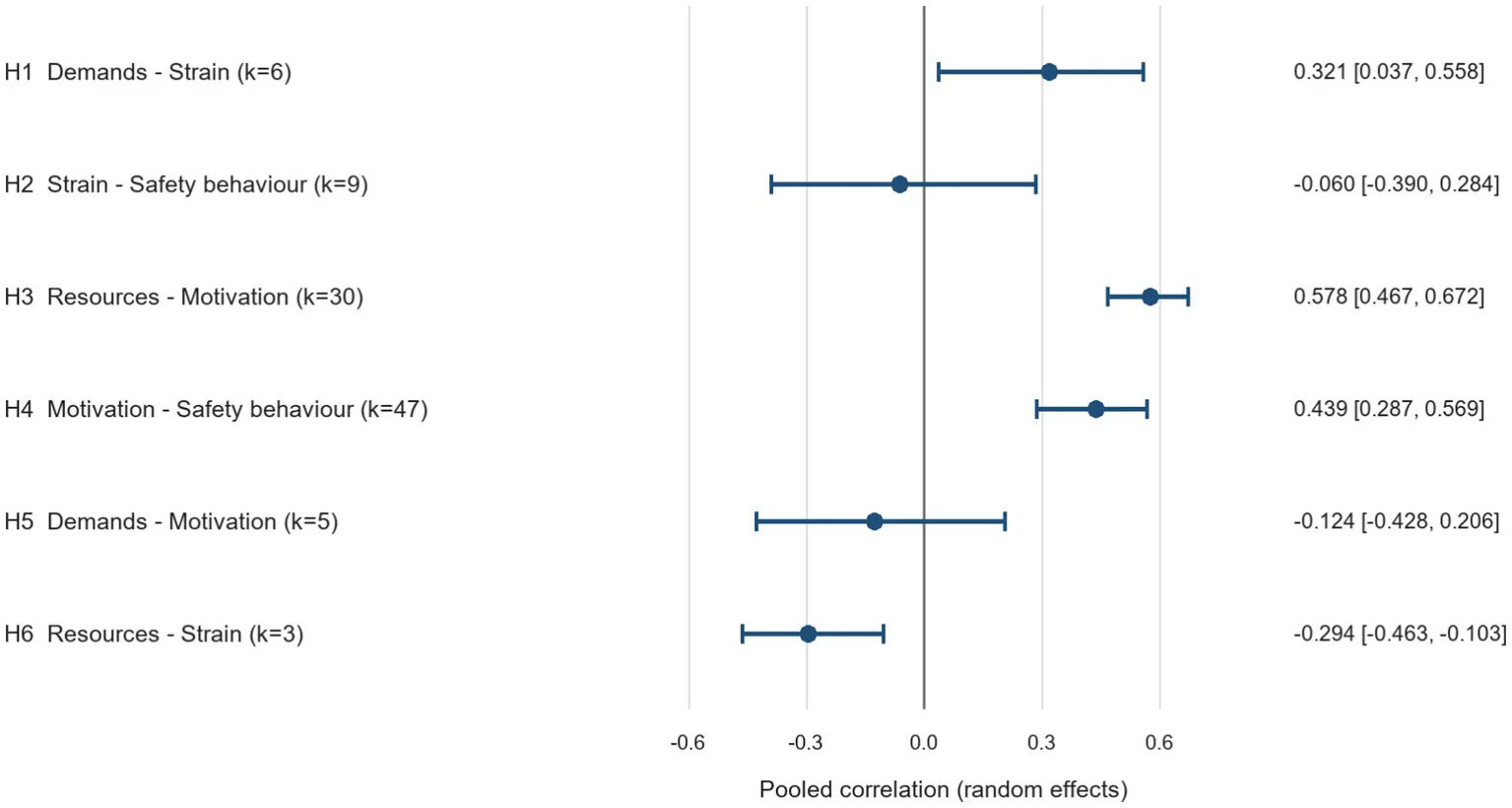
Forest plot of the six JD-R path estimates. H5 and H6 are interpreted descriptively because each is based on no more than five independent samples.

### 4.3 Heat-moderation hypotheses (H7–H10)

No eligible high-heat subgroup sample remained for the demands–strain path (H7) or the strain–safety behavior path (H9). The high-heat entries from the previously coded Gu report measured safety behavior intention rather than behavior and included near-perfect correlations; they were retained only in sensitivity analysis. H7 and H9 are, therefore, not testable and should not be interpreted as null.

The two testable subgroup comparisons did not detect moderation. For resources-motivation (H8), the high-heat estimate was *r* = 0.640 (*k* = 4), and the normal/mixed estimate was *r* = 0.569 (*k* = 26), Qb = 0.234, and *p* = 0.628. For motivation–safety behavior (H10), the high-heat estimate was *r* = 0.596 (*k* = 4), and the normal/mixed estimate was *r* = 0.422 (*k* = 43), Qb = 0.538, and *p* = 0.463. Both point estimates were numerically higher under high heat, contrary to the hypothesized weakening effects, but the contrasts were imprecise and non-significant (Figure 3 and Table 2).

**Figure 3:**
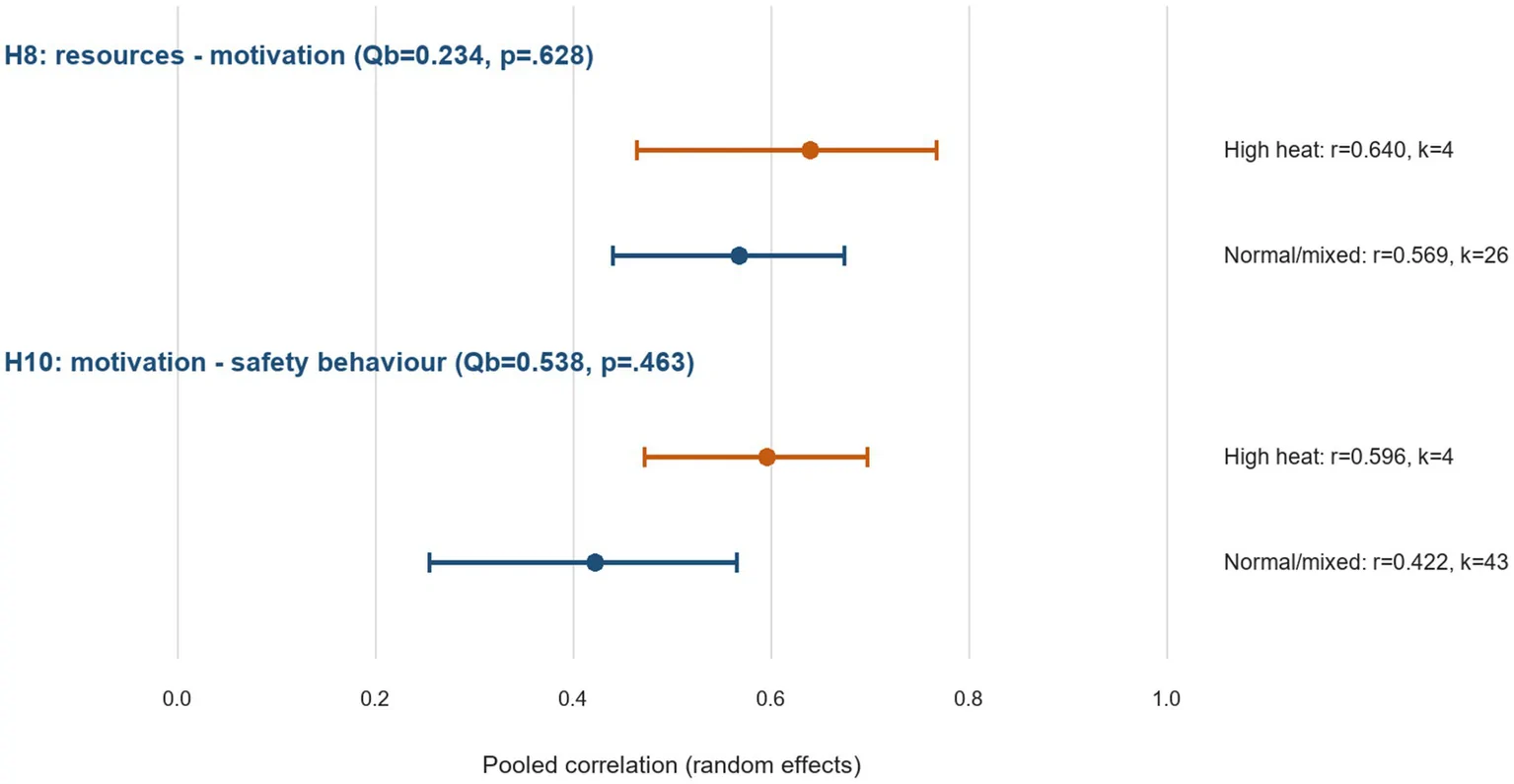
Testable heat-proxy subgroup estimates for H8 and H10.

**Table 2:** Heat-proxy subgroup analyses.

Path (H)	Subgroup	*k*	*N*	*r* [95% CI]	I^2^ (%)	Qb	Test status
H7	High heat	0	–	Not estimable	–	–	Not testable (0 eligible high-heat samples)
	Normal/mixed	6	2,532	0.321 [0.037, 0.558]	98.2	–	
H8	High heat	4	1,427	0.640 [0.465, 0.767]	95.6	0.234	Tested; n.s. (*p* = 0.628)
	Normal/mixed	26	9,167	0.569 [0.440, 0.674]	98.6	–	
H9	High heat	0	–	Not estimable	–	–	Not testable (0 eligible high-heat samples)
	Normal/mixed	9	3,195	−0.060 [−0.390, 0.284]	99.0	–	
H10	High heat	4	1,527	0.596 [0.472, 0.698]	91.0	0.538	Tested; n.s. (*p* = 0.463)
	Normal/mixed	43	14,824	0.422 [0.255, 0.566]	99.3	–	

H7 and H9 are not testable because the eligible high-heat subgroup is empty.

### 4.4 Publication bias and small-study effects

Egger regression analysis was conducted only for H3 (*k* = 30) and H4 (*k* = 47). Paths with k less than 10 were not tested because asymmetry tests have low power and may have unstable error rates in small sets.

Neither Egger intercept was statistically significant (H3: intercept = 7.087, *t* = 0.893, *p* = 0.379; H4: intercept = 6.040, *t* = 0.743, *p* = 0.461). These tests do not prove the absence of reporting bias, particularly under extreme heterogeneity, but they provide no evidence of detected funnel asymmetry. Figure 4 presents descriptive funnel plots.

**Figure 4:**
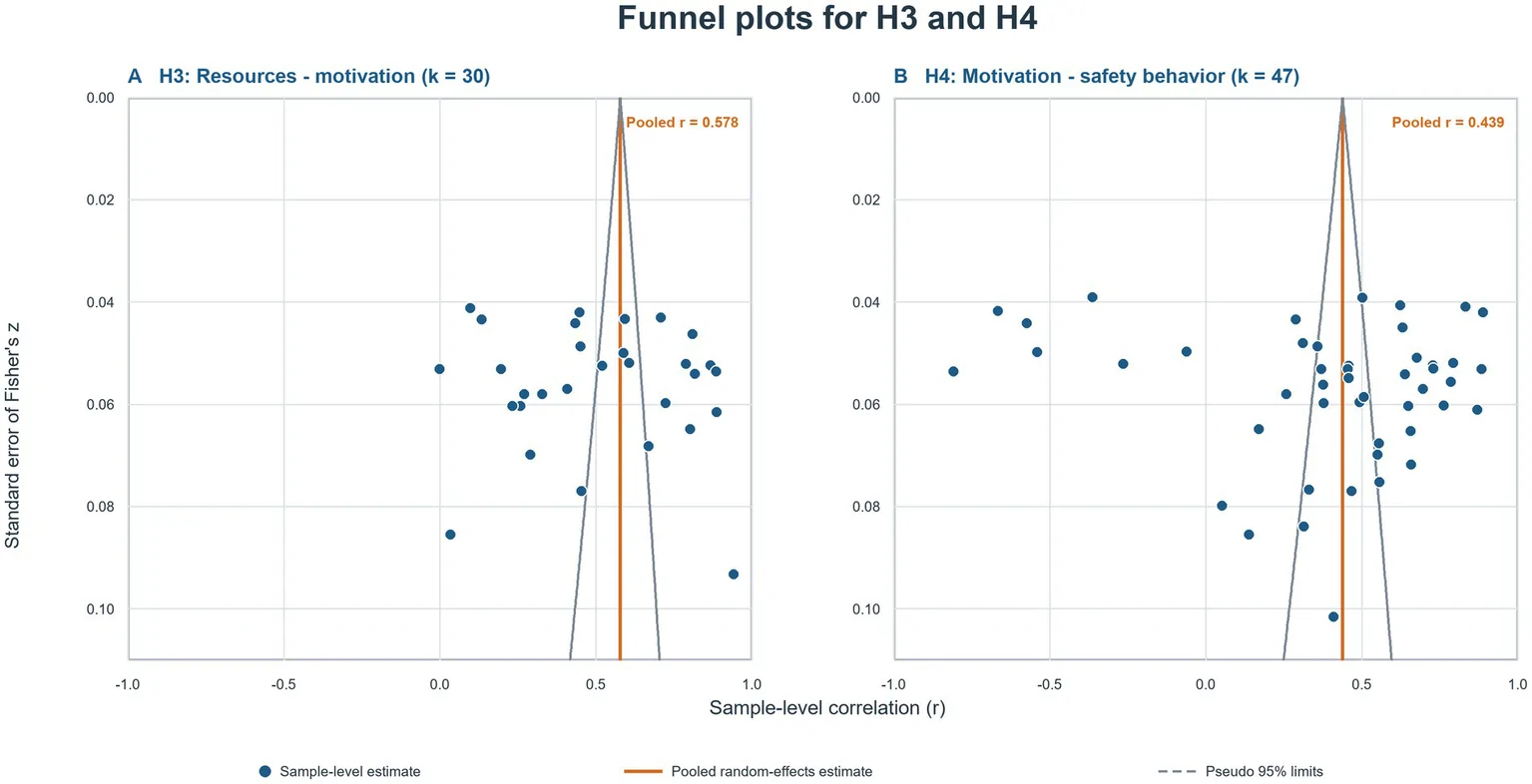
Funnel plots for H3 and H4. The funnel shape is descriptive because heterogeneity is substantial.

### 4.5 Sensitivity analyses and construct consistency

The inclusive primary mapping retained defensible boundary constructs to avoid an unnecessarily conservative evidence base. A restricted rule-based construct mapping produced nearly identical H3 and H4 estimates (*r* = 0.580 and 0.433). Removing correlations with |*r*| > 0.85 reduced their magnitudes but not their direction or statistical significance (*r* = 0.474 and 0.383). Treating safety behavior intention as a proxy outcome changed H2 to *r* = −0.263 but left its interval wide and non-significant; H4 remained positive (*r* = 0.464). These comparisons show that the motivational findings are not artifacts of the inclusive mapping, whereas H2 remains definition-sensitive and heterogeneous.

Safety compliance and safety participation were combined as facets of safety behavior because a dependence-robust, within-sample comparison detected no significant difference (CR2 within-sample coefficient = −0.008, df = 13.586, *p* = 0.885); a paired random-effects sensitivity analysis was also non-significant (Delta *z* = 0.035, *p* = 0.411). This is not an equivalence test: the analysis supports the decision to pool the facets for the present synthesis but does not establish that they are interchangeable in all contexts.

## 5 Discussion

### 5.1 Theoretical implications and JD-R processes in construction work

The clearest theoretical result is the robustness of the motivational process. Resources were strongly associated with motivation (H3: *r* = 0.578), and motivation was associated with safety behavior (H4: *r* = 0.439), consistent with JD-R theory and prior safety meta-analyses (5, 6). The contribution beyond applying JD-R to another occupational domain is twofold. First, the analysis shows that the resource–motivation–behavior sequence generalizes across construction work samples, including substantial evidence from China, even after dependence, construct boundary, and influential-effect corrections. Second, it separates the conceptual role of heat as a physical demand from its empirical role as a boundary condition. The former is theoretically plausible; the latter remains unestablished because eligible high-heat psychological samples are scarce.

The impairment process was less stable. Demands were positively related to strain (H1: *r* = 0.321), but the strain–safety behavior association was unresolved (H2: *r* = −0.060; I^2^ = 99.0%). This differs from Nahrgang et al.’s (6) study, which reported a clearer negative burnout-safety association. Our broader strain domain includes fatigue, anxiety, negative affect, symptoms, and cognitive dissonance, while outcome orientation and self-report practices vary across studies. The wide interval, rather than the near-zero point estimate alone, is the substantive finding: the current construction evidence cannot identify a common H2 magnitude.

The cross-links should not be overinterpreted. H5 was negative but imprecise, consistent with mixed challenge and hindrance demands. H6 was negative in the expected buffering direction, but only three samples contributed. These estimates correct the earlier, path-misaligned H5/H6 pools and remove the previous speculative explanation of a positive resource-strain association. Their main theoretical value is to identify where primary evidence is missing.

### 5.2 Moderator analyses: resource type and extreme heat

Construct and design heterogeneity is substantive rather than a nuisance that random effects alone can eliminate. Safety compliance and participation did not differ detectably after accounting for shared samples, supporting their joint use here. In contrast, occupation and regional labels were too fragmented for stable moderator tests; measurement scales were not consistently archived; and only two H4 samples were longitudinal. Thus, high I^2^ may reflect strain type, resource operationalization, reporting orientation, occupation, climate, and design, but the available coding cannot apportion their relative contributions. This limitation is explicitly separated from evidence of no moderation.

Extreme heat was not established as a moderator. H7 and H9 had no eligible high-heat samples, and H8/H10 showed non-significant subgroup differences. The point estimates for H8 and H10 were numerically larger under the heat proxy, contrary to the proposed weakening mechanism. This pattern could reflect greater reliance on motivational resources in difficult conditions, but it could equally reflect regional, seasonal, or measurement differences. The data do not discriminate among these explanations.

Accordingly, the result is an absence of adequate evidence, not evidence of absence. A non-significant Qb with four high-heat samples does not demonstrate equivalence, and a season/region proxy is not interchangeable with measured WBGT. The most defensible conclusion is that heat remains a plausible physical demand and candidate boundary condition whose psychological moderation effects require purpose-designed primary studies.

This conclusion also reconciles apparently inconsistent prior evidence. Physiological and epidemiological research has reliably linked heat to fatigue, cognitive impairment, injury, and productivity loss, whereas the present individual-level JD-R correlations do not detect path moderation. The difference reflects levels of analysis and measurement, not necessarily a substantive contradiction. Direct heat-outcome studies answer whether heat is hazardous; the present subgroup tests ask whether heat changes correlations among psychological constructs.

### 5.3 Practical implications

First, job resources should be strengthened to support motivation: supervisory support, credible safety communication, worker participation, recognition, and access to usable protective resources. The stable H3/H4 findings justify this recommendation across the pooled settings, while the heat subgroup analysis does not establish that these resources become weaker under hot conditions.

Second, heat should be managed as a direct physical hazard rather than waiting for evidence of psychological moderation. Sites should monitor WBGT, provide hydration and shaded recovery areas, schedule work–rest cycles, rotate demanding tasks, and empower supervisors to slow or stop work. These controls are supported by the occupational-heat literature; they are not claimed as causal consequences of the non-significant H8/H10 tests.

For resource allocation, the motivational process is currently more reproducible than the impairment-to-behavior link. Managers can act on that evidence while treating H2, H5, H6, and heat moderation as priorities for better primary measurement rather than as settled null or positive effects.

### 5.4 Limitations

Several limitations bear emphasis. First, original database exports, exact historical search dates, and record-level search counts were not archived. The revision provides the exact reconstructed strings and all recoverable corpus counts but cannot convert an inherited evidence inventory into a fully prospective PRISMA search record. Second, heterogeneity was extreme, meaning that pooled values average across diverse constructs, scales, occupations, and study designs. Third, boundary constructs were adjudicated using an inclusive rule and therefore require sensitivity analysis; the restricted and influential-effect analyses partly address, but do not remove, this judgment.

H1, H5, and H6 contain only six, five, and three samples, respectively. H5 and H6 should be read as descriptive. H2 also has only nine samples and remains highly definition-sensitive. The motivational findings are better populated but still heterogeneous.

The heat indicator is a season/region proxy rather than measured WBGT and is confounded with place, season, and study design. High altitude was not coded as high heat. Behavioral-intention outcomes and the near-perfect correlations in the previously coded Gu report were excluded from the primary analysis, leaving no eligible high-heat sample for H9. Mo and Jiang (17) classified the high-heat sample as normal/mixed and cognitive dissonance as strain based on the verified full text.

The majority of coded studies were cross-sectional, so causal direction cannot be inferred. Occupation, scale, and longitudinal-design moderation could not be tested adequately because these fields were sparse or unbalanced. Future updates should prospectively register the protocol, export every database result with a timestamp, double-screen at the report level, and extract scale, occupation, design, and objective heat exposure before effect-size coding.

### 5.5 Future research

Three priorities emerge from this synthesis.

First, primary studies should measure JD-R constructs and safety behavior alongside objective heat exposure (e.g., WBGT) at the individual level, rather than relying on season or region proxies. Recent research has demonstrated the feasibility of combining wearable physiological monitoring with WBGT measurements in construction settings (13, 21). Ecological momentary assessment (EMA) using wearable sensors for temperature and heart rate, combined with brief smartphone surveys on demands, resources, strain, and motivation, would be ideal (4, 7). Such designs could capture within-person variation across different ambient temperatures. This would allow demands → strain and strain → safety behavior links to be estimated within genuinely high-heat samples.

Second, longitudinal and diary designs are needed to separate heat effects from seasonal and regional confounds. Cross-sectional designs cannot distinguish whether observed high-heat differences reflect temperature, sampling, or regional management practices. Evidence from longitudinal studies on heat and cognitive performance (13, 22) suggests that cumulative heat exposure over several days can have delayed and persistent effects. Repeated measures over summer and winter months, or across regions with different climates, would help isolate the causal effect of heat.

Third, pre-registered moderation tests with adequate statistical power require more high-heat studies. Stanley et al. (23) showed that meta-analyses with low median retrospective power (<50%) are prone to false positives and that pre-registration substantially increases the credibility of replication. For categorical moderator tests in meta-analysis, power depends on the number of studies per subgroup, between-study variance, and the magnitude of the moderation effect (24). Researchers should aim for high-heat subsamples of at least *k* ≥ 5 to enable meaningful subgroup comparisons. A realistic target would be to conduct multi-site studies in southern China during summer, where WBGT regularly exceeds 28 °C, and collect JD-R data on at least 200 workers per site.

Until such evidence accumulates, the proposition that extreme heat reshapes JD-R safety processes remains a working hypothesis rather than an established finding.

## 6 Conclusion

This meta-analysis indicates that the motivational JD-R process is the most reproducible pathway in construction safety: job resources are associated with motivation, and motivation is associated with safer behavior. Demands are associated with strain, but the strain-behavior link remains unresolved. The cross-links are based on too few samples for firm conclusions.

The available evidence does not establish extreme heat as a moderator. H7 and H9 are not testable after construct-consistent screening, and H8/H10 show no detected subgroup difference. This is an evidence gap, not proof of no effect. Heat should still be managed as a direct occupational hazard using objective exposure monitoring and physical controls.

The practical priority is therefore twofold: strengthen motivational resources such as supportive supervision and safety climate, and independently implement heat controls such as hydration, shade, task rotation, and work–rest cycles. The revised dataset, decision log, sample crosswalk, analysis code, and retrospective revision-stage plan provide a transparent basis for future prospectively registered updates.

## Data Availability

The original contributions presented in the study are included in the article/Supplementary material, further inquiries can be directed to the corresponding author.
