# Supplementary material for: The job demands–resources model and safety behavior in construction work: a systematic review and meta-analysis of six dual-process paths
Source: Front Public Health. 2026 Sep 23;14:1938727. doi: 10.3389/fpubh.2026.1938727 (PMC13643690; doi:10.3389/fpubh.2026.1938727)
Supplement: Supplementary file 1 [file Supplementary_file_1.docx]

*Supplementary Materials*

# S1. Revision-stage analysis plan and registration status

Registration status. This document is a retrospective revision-stage analysis amendment, not a prospective preregistration. The original review began from an inherited coding workbook and no external protocol was registered before the original screening or extraction. The amendment was frozen before the present final reanalysis to reduce flexibility and to make deviations auditable.

## Decisions frozen for the revision

1. Use zero-order correlations and orient unsafe-behaviour outcomes so higher values indicate safer behaviour.
2. Assign paths from the actual construct pair, accepting reversed x/y order because correlations are symmetric.
3. Pool multiple correlations within each underlying sample and path by averaging Fisher's z.
4. Separate independent samples within reports and cluster publications sharing a global sample identifier.
5. Use an inclusive primary construct mapping when theoretically defensible; compare it with a restricted rule-based construct mapping.
6. Exclude behavioural intention, incidents/near misses, and outcome-outcome correlations from the primary safety-behaviour pool; retain intention as sensitivity only.
7. Quarantine |r| >= .99 and run an influential-effect sensitivity excluding |r| > .85.
8. Classify cognitive dissonance as strain; classify high altitude as normal/mixed rather than high heat.
9. Require at least two independent samples in each heat subgroup before interpreting Qb.
10. Treat H5/H6 estimates with k < 5 as descriptive regardless of nominal p values.

## Departures discovered during verification

Long et al. (2026) and Li et al. (2026) contained usable zero-order correlation tables and were added after full-text verification. The original H5/H6 labels contained off-path records and were rebuilt. These departures are additions prompted by source verification, not outcome-driven exclusions.

# S2. Search reconstruction and corpus provenance

The historical searches were reported as using Web of Science, Scopus, CNKI, and Wanfang. Exact database exports, execution dates, and record-level search counts were not preserved. The strings below are reconstructed from the coding workbook, manuscript, and retained search terms so that a future update can be rerun; they are not represented as contemporaneously archived original queries. English- and Chinese-language publications were eligible, with no geographic restriction.

| **Database** | **Field / reconstructed query** | **Language and date record** |
| --- | --- | --- |
| Web of Science Core Collection | TS=(("construction worker*" OR "construction personnel" OR "building worker*" OR "construction labor*") AND ("safety behavio?r" OR "unsafe behavio?r" OR "safety compliance" OR "safety participation") AND ("job demand*" OR "job resource*" OR strain OR burnout OR fatigue OR anxiety OR motivation OR engagement OR "safety climate" OR leadership OR support)) | English; original execution date unavailable |
| Scopus | TITLE-ABS-KEY(("construction worker*" OR "construction personnel" OR "building worker*" OR "construction labor*") AND ("safety behavio?r" OR "unsafe behavio?r" OR "safety compliance" OR "safety participation") AND ("job demand*" OR "job resource*" OR strain OR burnout OR fatigue OR anxiety OR motivation OR engagement OR "safety climate" OR leadership OR support)) | English; original execution date unavailable |
| CNKI | 主题=(建筑工人 OR 施工人员 OR 建筑业工人) AND (安全行为 OR 不安全行为 OR 安全遵守 OR 安全参与) AND (工作要求 OR 工作资源 OR 压力 OR 倦怠 OR 疲劳 OR 焦虑 OR 安全动机 OR 工作投入 OR 安全氛围 OR 安全领导 OR 社会支持) | Chinese; original execution date unavailable |
| Wanfang Data | 主题=(建筑工人 OR 施工人员 OR 建筑业工人) AND (安全行为 OR 不安全行为 OR 安全遵守 OR 安全参与) AND (工作要求 OR 工作资源 OR 压力 OR 倦怠 OR 疲劳 OR 焦虑 OR 安全动机 OR 工作投入 OR 安全氛围 OR 安全领导 OR 社会支持) | Chinese; original execution date unavailable |

## S2.1. Recoverable report-level corpus reconciliation

The report-level quantities below are derived from the surviving coding workbook and the final record-level decision log. They do not replace the unavailable historical database-identification and title/abstract-screening counts. During revision, nine specifically named candidate full texts were verified. Two of these (Hu et al. and Huang & Li) were already represented among the 69 inherited reports, while the remaining seven were external candidates. This nine-report verification set is a revision-stage source check and should not be confused with the eight 2025-2026 reports already retained in the inherited analytical file. The inherited analytical file also contains a separate report labelled Li, 2026 (N = 407). It is not the N = 425 AI-adoption report added during full-text verification; Supplementary Data S1-S3 distinguish them as RS_Li, 2026 and LI_2026_AI, respectively.

| **Report-level stage** | **n** | **Evidence / interpretation** |
| --- | --- | --- |
| Historical database identification, deduplication, and title/abstract screening | Unavailable | Original exports, dates, and screening logs were not archived |
| Inherited report blocks | 117 | Recoverable coding workbook |
| Represented in the surviving 255-row inherited effect-size file | 69 | Unique inherited report identifiers in Supplementary Data S1 |
| Not represented in the surviving inherited effect-size file | 48 | 117 - 69; original report-level dispositions not fully archived |
| Represented reports retained after revision-stage adjudication | 60 | 52 pre-update reports plus eight 2025-2026 reports |
| Represented reports with no eligible primary six-path contribution | 9 | Off-path, outcome-only, or intention-only records after construct/path review |
| External candidate full texts assessed during revision | 7 | Candidates outside the inherited 69-report effect-size file |
| External candidates added to the primary synthesis | 3 | Li et al. (2026; N=425; 10 correlations), Long et al. (2026; N=208; 6 correlations), and Mo and Jiang (2026; N=488; 4 correlations) |
| External candidates not contributing to the primary synthesis | 4 | Alahmad et al. (2025), Marchiori and Song (2025), Wu et al. (2026), and Zailani et al. (2026) |
| Final included reports | 63 | 60 retained inherited reports plus three additions |

## S2.2. Effect-size audit

| **Effect-size stage** | **n** | **Decision** |
| --- | --- | --- |
| Coded effect-size database | 343 | Starting analytical inventory |
| After exact-duplicate removal | 275 | 68 exact duplicate rows removed |
| After original eligibility exclusions | 255 | 20 removed: 6 ecological, 10 heat-as-predictor, and 4 unverifiable-source entries |
| After revision construct/path adjudication | 174 | 81 inherited entries excluded or quarantined |
| File-verified additions | 20 | Eligible correlations from three newly added reports |
| Final primary synthesis | 194 | 174 inherited plus 20 file-verified additions |

# S3. Construct and outcome decision rules

| **Construct / outcome** | **Primary decision** | **Rationale** |
| --- | --- | --- |
| Safety compliance; safety participation | Pool as safety behaviour facets | Dependence-robust comparison detected no significant difference; not claimed equivalent |
| Unsafe behaviour | Retain with sign reversal | Creates a common higher-is-safer orientation |
| Safety-behaviour intention | Exclude primary; include sensitivity | Intention is an antecedent, not enacted behaviour |
| Incidents; near misses | Exclude | Safety outcomes are not safety-behaviour measures |
| Safety performance | Retain only if behaviour-based | Avoid mixing accident outcomes with behavioural scales |
| PBC | Map to motivation when scale content supports control belief | PBC is not safety behaviour |
| Safety knowledge / ability | Personal resource | Capacity that supports goal attainment |
| Safety attitude / responsibility / thriving / relational energy | Motivation | Motivational or engagement-based state |
| Li et al. (2026): AI adoption | Job demand | Scale captures the extent of required AI use and time spent working with AI in core job functions |
| Li et al. (2026): AI usefulness / adaptability | Resource side | Task-enabling support and individual capacity to adjust to changing work requirements |
| Li et al. (2026): safety responsibility / relational energy | Motivation | Felt safety obligation and a motivational psychological state generated through interaction |
| Li et al. (2026): extra-role safety behaviour | Safety participation | Voluntary effort to improve worksite safety beyond mandatory role requirements |
| Cognitive dissonance | Strain | Verified source defines an aversive resource-consuming state |
| High altitude | Normal/mixed heat group | Altitude is not evidence of high ambient heat |

# S4. Main and sensitivity results

| **Path** | **k** | **N** | **r** | **95% CI** | **p** | **I2** | **tau2** |
| --- | --- | --- | --- | --- | --- | --- | --- |
| H1 | 6 | 2532 | 0.321 | [0.037, 0.558] | 0.027 | 98.2 | 0.1342 |
| H2 | 9 | 3195 | -0.060 | [-0.390, 0.284] | 0.739 | 99.0 | 0.2867 |
| H3 | 30 | 10594 | 0.578 | [0.467, 0.672] | <.001 | 98.4 | 0.1810 |
| H4 | 47 | 16351 | 0.439 | [0.287, 0.569] | <.001 | 99.2 | 0.3716 |
| H5 | 5 | 1551 | -0.124 | [-0.428, 0.206] | 0.464 | 97.7 | 0.1406 |
| H6 | 3 | 1458 | -0.294 | [-0.463, -0.103] | 0.003 | 92.4 | 0.0283 |

## S4.1. Sensitivity analyses

| **Scenario** | **Path** | **k** | **r** | **95% CI** | **p** |
| --- | --- | --- | --- | --- | --- |
| primary_inclusive | H1 | 6 | 0.321 | [0.037, 0.558] | 0.027 |
| primary_inclusive | H2 | 9 | -0.060 | [-0.390, 0.284] | 0.739 |
| primary_inclusive | H3 | 30 | 0.578 | [0.467, 0.672] | <.001 |
| primary_inclusive | H4 | 47 | 0.439 | [0.287, 0.569] | <.001 |
| primary_inclusive | H5 | 5 | -0.124 | [-0.428, 0.206] | 0.464 |
| primary_inclusive | H6 | 3 | -0.294 | [-0.463, -0.103] | 0.003 |
| restricted_rule_based_mapping | H1 | 6 | 0.321 | [0.037, 0.558] | 0.027 |
| restricted_rule_based_mapping | H2 | 9 | -0.060 | [-0.390, 0.284] | 0.739 |
| restricted_rule_based_mapping | H3 | 27 | 0.580 | [0.462, 0.679] | <.001 |
| restricted_rule_based_mapping | H4 | 41 | 0.433 | [0.267, 0.573] | <.001 |
| restricted_rule_based_mapping | H5 | 2 | -0.267 | [-0.338, -0.193] | <.001 |
| restricted_rule_based_mapping | H6 | 2 | -0.352 | [-0.534, -0.138] | 0.002 |
| exclude_abs_r_gt_0.85 | H1 | 6 | 0.321 | [0.037, 0.558] | 0.027 |
| exclude_abs_r_gt_0.85 | H2 | 9 | -0.060 | [-0.390, 0.284] | 0.739 |
| exclude_abs_r_gt_0.85 | H3 | 26 | 0.474 | [0.370, 0.566] | <.001 |
| exclude_abs_r_gt_0.85 | H4 | 44 | 0.383 | [0.232, 0.516] | <.001 |
| exclude_abs_r_gt_0.85 | H5 | 5 | -0.124 | [-0.428, 0.206] | 0.464 |
| exclude_abs_r_gt_0.85 | H6 | 3 | -0.294 | [-0.463, -0.103] | 0.003 |
| include_behavioural_intention_proxy | H1 | 6 | 0.321 | [0.037, 0.558] | 0.027 |
| include_behavioural_intention_proxy | H2 | 10 | -0.263 | [-0.672, 0.268] | 0.331 |
| include_behavioural_intention_proxy | H3 | 30 | 0.578 | [0.467, 0.672] | <.001 |
| include_behavioural_intention_proxy | H4 | 48 | 0.464 | [0.311, 0.594] | <.001 |
| include_behavioural_intention_proxy | H5 | 5 | -0.124 | [-0.428, 0.206] | 0.464 |
| include_behavioural_intention_proxy | H6 | 3 | -0.294 | [-0.463, -0.103] | 0.003 |

## S4a. Dependence-robust comparison of safety-behaviour facets

Participation was coded 1 and compliance 0, so positive coefficients indicate higher correlations for participation. The primary CR2 model separated within-sample from between-sample variation and clustered effects by underlying sample. The paired sensitivity compared participation and compliance correlations for the same predictor within the same sample, using a random-effects model with the within-pair correlation set to rho = .50.

| **Dataset and model** | **Estimate** | **SE** | **df / k** | **p** | **Evidence base** |
| --- | --- | --- | --- | --- | --- |
| All eligible facets: within-sample CR2 | b = -0.0078 | 0.0529 | df = 13.586 | 0.885 | 68 effects; 22 sample clusters |
| All eligible facets: paired random effects | Delta z = 0.0350 | 0.0426 | k = 18 samples | 0.411 | 28 matched predictor-facet pairs |
| Restricted mapping: within-sample CR2 | b = -0.0290 | 0.0607 | df = 10.885 | 0.642 | 54 effects; 18 sample clusters |
| Restricted mapping: paired random effects | Delta z = 0.0101 | 0.0427 | k = 15 samples | 0.813 | 24 matched predictor-facet pairs |

Neither analysis detected a facet difference. These results support pooling for the present synthesis but do not establish equivalence between compliance and participation.

# S5. Independent samples and path contributions

| **Underlying sample** | **Report(s)** | **N** | **Path(s)** | **Correlations** |
| --- | --- | --- | --- | --- |
| LI_2026_AI | Li et al., 2026 | 425 | H3, H4, H5 | 10 |
| LONG_2026 | Long et al., 2026 | 208 | H1, H2, H3, H4, H5, H6 | 6 |
| MO_JIANG_2026 | Mo & Jiang, 2026 | 488 | H2, H6 | 4 |
| RS_Dera, 2025 | Dera, 2025 | 366 | H3, H4 | 3 |
| RS_Gao, 2025 | Gao, 2025 | 160 | H2, H4, H5 | 13 |
| RS_Hu, 2025 | Hu, 2025 | 762 | H1, H6 | 4 |
| RS_Li, 2026 | Li, 2026 | 407 | H4 | 1 |
| RS_Liu, 2025 | Liu, 2025 | 436 | H4, H5 | 12 |
| RS_Seo, 2026 | Seo, 2026 | 300 | H3, H4 | 4 |
| RS_Tang, 2026 | Tang, 2026 | 601 | H4 | 2 |
| RS_Wang, 2025 | Wang, 2025 | 283 | H3, H4 | 3 |
| WB_S1 | zheng,2021 | 535 | H3, H4 | 6 |
| WB_S100 | meng,2022 | 197 | H4 | 1 |
| WB_S101 | meng,2022 | 180 | H4 | 1 |
| WB_S11 | wang,2021 | 389 | H4 | 4 |
| WB_S12 | newaz,2021 | 374 | H4 | 2 |
| WB_S14 | Li,2021 | 357 | H2, H3, H4 | 4 |
| WB_S15 | He,2019; He,2021a | 655 | H3, H4 | 8 |
| WB_S17 | 王倩云,2021 | 279 | H4 | 1 |
| WB_S18 | 倪国栋,2021 | 609 | H4 | 4 |
| WB_S19 | 陈伟柯,2021 | 357 | H3, H4 | 3 |
| WB_S2 | ye,2022 | 335 | H1, H2, H4 | 7 |
| WB_S20 | xia,2020 | 311 | H4 | 2 |
| WB_S21 | ni,2020 | 368 | H3, H4 | 5 |
| WB_S23 | 伍娜,2020 | 358 | H4 | 2 |
| WB_S24 | liu q,2020 | 285 | H4 | 1 |
| WB_S26 | 孙剑,2019 | 241 | H3, H4 | 3 |
| WB_S27 | 彭川宇,2019 | 470 | H3 | 1 |
| WB_S3 | xie,2022 | 172 | H3, H4 | 7 |
| WB_S31 | 高静,2018 | 354 | H2 | 2 |
| WB_S32 | liao,2017 | 100 | H4 | 3 |
| WB_S33 | liao,2017 | 145 | H4 | 3 |
| WB_S34 | 杨高升,2017 | 140 | H3, H4 | 4 |
| WB_S39 | 赵富强,2015 | 578 | H1, H2, H4 | 3 |
| WB_S40 | 董小刚,2015 | 660 | H4 | 1 |
| WB_S43 | 董小刚,2014a; 董小刚,2014b | 408 | H4 | 3 |
| WB_S46 | siu,2004 | 374 | H3 | 1 |
| WB_S48 | Zhang J,2020 | 311 | H3 | 2 |
| WB_S5 | Liang h,2022 | 498 | H4 | 1 |
| WB_S50 | liang q,2022 | 517 | H3, H4 | 2 |
| WB_S52 | chen,2020 | 349 | H1 | 1 |
| WB_S53 | 史玉芳,2020a | 344 | H2, H4 | 4 |
| WB_S54 | 史玉芳,2020b | 218 | H3 | 1 |
| WB_S56 | 杨高升,2019 | 320 | H4 | 1 |
| WB_S57 | liang,2018 | 345 | H3 | 1 |
| WB_S59 | ju,2016 | 592 | H3 | 3 |
| WB_S60 | wang,2018 | 359 | H4 | 2 |
| WB_S62 | 康梦月，2022 | 351 | H3, H4 | 2 |
| WB_S63 | 陈彦霖,2022 | 222 | H4 | 2 |
| WB_S65 | 陈梅，2021 | 371 | H2, H3, H4 | 3 |
| WB_S66 | han,2020 | 326 | H4 | 2 |
| WB_S68 | 姚明亮,2019b | 278 | H3 | 1 |
| WB_S69 | 王丹,2020 | 278 | H3, H4 | 2 |
| WB_S70 | 张力,2019 | 118 | H3 | 1 |
| WB_S71 | 成家磊,2017 | 267 | H3 | 1 |
| WB_S72 | 来延肖,2019 | 173 | H4 | 1 |
| WB_S74 | 祁神军,2018a | 322 | H5 | 1 |
| WB_S79 | 叶贵,2016 | 295 | H4 | 2 |
| WB_S85 | yuan,2015 | 238 | H4 | 2 |
| WB_S92 | zhou,2011 | 543 | H3 | 3 |
| WB_S93 | zhou,2011 | 404 | H3 | 3 |
| WB_S94 | 周建亮,2022 | 300 | H1, H3 | 3 |
| WB_S96 | Chen,2022 | 271 | H4 | 1 |
| WB_S99 | hu,2022 | 569 | H3, H4 | 2 |

Identifier note. LI_2026_AI identifies the N = 425 report by Li et al. (2026) on AI adoption; the suffix 'AI' denotes the study topic and does not indicate AI-generated content. RS_Li, 2026 identifies a different inherited report with N = 407. These labels are unique analytical sample identifiers, not reference-list year suffixes.

## S5a. Study-quality and risk-of-bias considerations

No formal report-level risk-of-bias worksheet survived for the inherited corpus, and full texts were not available for every inherited report during the revision. In the recoverable workbook, 102 of 121 report-sample records were labelled cross-sectional, five longitudinal, and 14 had no archived design label. The evidence is therefore treated as associational and vulnerable to common-method, temporal-order, and selective-reporting bias. Small k and extreme heterogeneity further reduce confidence for H1, H2, H5, and H6. A future prospectively registered update should retrieve every full text and apply a design-appropriate appraisal independently by two reviewers.

# S6. Update-candidate full-text decisions

| **Report** | **Decision** | **Reason / extracted contribution** |
| --- | --- | --- |
| Alahmad et al. (2025) | Exclude from JD-R paths | Direct heat/productivity and physiological exposure design; no eligible psychological path correlation |
| Hu et al. (2025) | Retain eligible paths | Work conditions-symptoms and symptoms-resilience correlations retained under inclusive mapping |
| Huang & Li (2026) | No six-path contribution | Time pressure-safety voice/behaviour pairs instantiate demand-behaviour or outcome intercorrelations |
| Li et al. (2026) | Retain H3/H4/H5 | N=425; Table 2 zero-order correlations. Original definitions support AI adoption as demand; AI usefulness/adaptability as resource-side constructs; safety responsibility/relational energy as motivation; extra-role safety behaviour as participation |
| Long et al. (2026) | Retain H1-H6 | N=208; Table 3 contains one eligible correlation for each path |
| Marchiori & Song (2025) | Exclude from JD-R paths | Experimental heat/fatigue design; heat is predictor |
| Mo & Jiang (2026) | Retain H2/H6 | N=488; cognitive dissonance and burnout mapped to strain; high altitude not high heat |
| Wu et al. (2026) | No six-path contribution | Attentional-bias scale development without eligible JD-R-to-safety-behaviour correlation |
| Zailani et al. (2026) | Sensitivity only / exclude primary | Outcome is safety behavioural intention |

# S7. Publication-bias diagnostics

| **Path** | **k** | **Egger intercept** | **t** | **df** | **p** |
| --- | --- | --- | --- | --- | --- |
| H3 | 30 | 7.087 | 0.893 | 28 | 0.379 |
| H4 | 47 | 6.040 | 0.743 | 45 | 0.461 |

No Egger intercept was statistically significant. Because heterogeneity is extreme, these results should not be read as proof that reporting bias is absent.

# S8. Record-level adjudication file

Supplementary Data S1 contains 275 rows: 255 inherited records plus 20 file-verified additions. Each row records its source, constructs, orientation, reconstructed path, inclusion decision, and reason. Supplementary Data S2 contains the final sample-path estimates, Supplementary Data S3 contains the report-sample crosswalk, Supplementary Data S4 contains the facet-analysis input, and Supplementary Code S1-S2 contain portable Python and R scripts used for the revised analyses.
